# Case of Gun Shot Wound of the Stomach

**Published:** 1856-08

**Authors:** Charles S. Tripler

**Affiliations:** Surgeon U. S. Army


### RECORD OF MEDICAL SCIENCE.

---

*Case of Gun Shot Wound of the Stomach.* By CHARLES S. TRIPLER, Surgeon U. S. Army.

In the aggregate number of wounds received in battle, it is fair to presume that no inconsiderable proportion will involve the stomach. When a General can choose his time for engaging the enemy, he will be careful to secure to his men a good meal before hand. Men, generally, go into action with the stomach well filled. Occupying, as it does under these circumstances, so large and so central a space in the body, it can hardly escape in the indiscriminate lesions consequent upon a well directed fire. And yet, few military surgeons have seen many cases. Hennen says he never treated one. Sir George Ballingall, though giving some most judicious observations upon the nature and treatment of these wounds, does not say that he had ever treated one. Larrey, in his campaigns in Russia and France, makes no mention of any. I am not in possession of his other volumes. Gibson reports no case in his surgery. Mr. Alcock, as quoted by Ballingall, saw but one recovery from a gun-shot wound or the stomach out of 3000 cases of gun-shot wounds. Thomson saw two recoveries after the battle of Waterloo.

There can be but one reason for all this—that is, that this lesion is almost invariable and speedily fatal. The stomach is so important an organ in its functions, in its relations, and its nervous connections, that it will rarely bear so severe an injury as that of a gun-shot wound. Hennen remarks, "Baron Percy calculates that out of twenty cases, four or five only have escaped; this, however, is a most favorable average." Sir G. Ballingall thinks Percy has abundant reasons to be satisfied with his success, and that the experience of others will hardly warrant us to expect a like result.

A just prognosis as to the issue of gun-shot wounds of the stomach cannot be deduced from the result of penetrating wounds from other causes, whether accidental, or due to operations for the extraction of foreign bodies. The circumstances are altogether different, and, in the latter case, time, place and other accidents can all be commanded. The gun-shot wound, on the other hand, partakes of the nature of a violent blow upon the stomach, a circumstance of itself frequently fatal, its extent is greater than most other penetrating wounds, its shape irregular, its situation as likely to be the most unfavorable as any other, and, in general, the time of its infliction will be when the stomach is distended with food. So, that it appears to me, that even when the sufferer reaches the hospital alive, the most unfavorable prognosis is the only prudent one in every case.

I have seen two cases of gun-shot wounds of the stomach. The first was a Mexican soldier, who fell into our hands at Enceno after the battle of Cerro Gordo. This poor fellow had a frightful wound from a musket ball toward the pyloric extremity of the stomach. He was in excruciating agony, with burning thirst, great prostration, but with the mind perfectly clear. Every drop of water given him, escaped immediately from the wound. There was no vomiting or retching in his case after I saw him. He lived nine hours from the time he was wounded.

The second case came under my care while I was serving in San Francisco, Ca.; being one of great interest and terminating favorably. I will now proceed to report it in detail.

On the 3d June, 1854, I was called to see my friend Dr. R. B. Cole who had accidentally shot himself. This gentleman was one of the proprietors of a superb apothecary's establishment, such as is seen only in San Francisco. He had been recently the subject of a tedious and exhausting malarial fever, was convalescent, and for change of air was about to make an excursion into the country for a few days. Whilst engaged in the back part of his store, in transferring some changes of clothing from one trunk to another, he took a loaded six-barrel pistol and thrust it carelessly, muzzle foremost, into the watch pocket of his vest. Then, as he stooped over again, the weight of the weapon caused it to fall from his pocket upon the marble floor, exploding one barrel, and the unfortunate gentleman fell, merely saying "I am shot." While the messenger ran for me, the Doctor was raised from the floor and carefully placed upon a sofa that was at hand. I saw him certainly within five minutes of the occurrence, probably less. He was pulseless, pupils dilated, no sign of respiration, no vibration even of the heart, lips blue, countenance colorless, body relaxed. I opened his shirt and found a wound over the cartilages of the seventh and eighth ribs on the left side within about an inch of the sternum. Introducing the point of my little finger, it passed obliquely upward, and became engaged in an irregular opening in the cartilage of the sixth rib. Not being then aware of the position of the patient at the time he received his wound, and judging from the relative direction of the perforation in the integument and that in the cartilage, I inferred the ball had passed upward, inward and backward, traversing the heart, and that the man was dead.

While asking some questions naturally suggested by the accident, I observed a faint effort at inspiration. I gave immediately a tea-spoonful of brandy, and had the pleasure of finding it was soon swallowed, and another feeble inspiration succeeded. By perseverance in the cautious use of brandy and ammònia, respiration was re-established, and in about ten or fifteen minutes, consciousness returned and with it the mind perfectly clear. Still, no pulse could be felt at the wrist—an occasional feeble and irregular fluttering of the heart could be detected.

By this time, quite a number of physicians had clustered about the patient. I had no little difficulty in preventing some of them from turning the wounded man over, to see where the ball had come out. It was obvious that the slightest motion under the circumstances must have caused a recurrence of syncope, and might have been fatal. I could not comprehend of what possible consequence it was, what had become of the ball, and I could not agree that the feeble flickering of life remaining should be extinguished for the gratification of an unscientific curiosity.

Drs. Bertody, Stout, Mott and Hewit, of San Francisco, all personal friends of the patient and of myself, were now present, and united with me in counsel and exertion to do whatever was rational or necessary in the case. These gentlemen most kindly assisted me throughout the treatment; for the first three nights and days, one of us was constantly in attendance, and subsequently at intervals of not to exceed a half hour until all danger had passed.

It had now become plain that the heart was not the seat of the internal lesion. What was its probable seat? The signs were those of nervous shock and hemorrhage, the prostration was extreme, the pulse continued feeble and fluttering, and frequently interrupted entirely for seconds at a time. We now knew from the patient himself how the accident occurred, and what was his position when he received the wound. His trunks were upon the floor,, and while he was stooping very far over, the pistol fell from his pocket. It was evident that, in this position, the wounded point of integument was in relation to a very different subjacent point of cartilage from that it occupied in the erect or supine position of the body; and that, therefore, the relative direction of the wound in the integument and that in the cartilage afforded no reliable indication of the true course of the ball. The only other independent element for determining this would have been the direction of the axis of the weapon, when it was discharged. This, of course, could not be ascertained, and the rational symptoms alone were left to guide our judgment. The possibility and probability of wound of the stomach early suggested itself, and the prognosis was, accordingly, most unfavorable.

By the cautions and persevering exhibition of stimulants, and maintaining absolute negation of muscular efforts, in about two hours, the heart's action was re-established though still very feeble and irregular. Any attempt to determine the number of pulsations would have been useless, as they were no two minutes alike.

The patient began to be restless and to feel some nausea. We were careful not to introduce a greater volume of fluid into the stomach than was absolutely necessary, as we feared the exhaustion to be expected from the exertion of vomiting. By dint of great effort on the part of both surgeon and patient, this was kept at bay for another half hour, when it could no longer be restrained. The patient turned his head aside, and while the body was supported, ejected from his stomach a coagulum of blood of about twelve ounces. The case was then solved. The stomach was the wounded organ. In what part of this was the probable seat of the injury?

Half an hour before the accident, he had eaten a light lunch not not exceeding four ounces in bulk. No fluid had escaped from the external wound. The hemorrhage into the stomach, which must have been instantaneous, was large. The position of the wound was far above the pyloric extremity of the stomach. The probabilities were therefore, that the wound was in the cardiac portion, and this was one circumstance in our favor. On the other hand, the fact of the stomach being wounded at all, the debilitated and irritable condition of the patient, the effect of antecedent disease, his nervous lymphatic temperament, his anxiety as to the result, aggravated by family responsibility and instructed by professional acquirement, all combined to suggest the most gloomy forebodings. The occurrence of hiccup a few minutes after the vomiting did not tend to quiet our apprehensions. Fortunately, this symptom soon subsided and gave us no further trouble throughout the case.

The plan of treatment adopted and rigidly adhered to, was perfect rest and quiet, the patient to be kept narcotized with Morphia, nothing to be taken into the stomach except the water in which the Morphia was dissolved and that from the small pieces of ice with which the mouth was cooled, nourishment, when required, to be by enemata of beef tea. It was not intended that any evacuation of the bowels should be solicited for several days. I remained with him that night, and gave him from a quarter to half a grain of Acetate of Morphia, whenever signs of restlessness appeared. He had quite a comfortable night, and by morning there was an encouraging improvement in all the signs. I left him at 9 A. M., June 4th, having just administered half a grain of Morphia in a drachm of water. From that time till June 12th inclusive, the following is a correct record of the quantities of Morphia and beef juice exhibited, and the time each portion was given.

| | | |
|---|---|---|
| June 4th, | 12.15 P. M. | Acet. Morph., gr. $\frac{1}{4}$. |
| " " | 2.5 " | " gr. $\frac{1}{2}$. |
| " " | 10.00 " | " " |
| " 5th, | 3.00 A. M. | " " |
| " " | 8.00 " | " gr. $\frac{1}{4}$. |
| " " | 2.30 P. M. | " gr. $\frac{1}{2}$. |
| " " | 8.00 " | " " |
| " " | 11.45 " | " " |
| " 6th, | 4.00 A. M. | " " |
| " " | 12.45 P. M. | Beef Tea, 2 ouuces by enema. |
| " " | 1.40 " | Acet. Morph., gr. $\frac{1}{2}$. |
| " " | 6.00 " | Beef Tea, 2 ounces, by enema. |
| " " | 10.40 " | Acet. Morph., gr. $\frac{1}{2}$. |
| " 7th, | 9.30 A. M. | " gr. $\frac{1}{4}$. |
| " " | 10.30 " | " " |
| " " | " " | Beef Tea, 2 ounces, by enema. |
| " " | 5.30 P. M. | " " " |
| " " | " " | Acet. Morph., gr. $\frac{1}{2}$. |
| " " | 10.00 " | " " |
| " 8th, | 2.30 A. M. | " " |
| " " | 12.30 P. M. | Beef Tea, 1 drach., by mouth. |
| " " | 2.30 " | " " " |
| " " | 5.30 " | " " " |
| " " | " " | Acet. Morph., gr. $\frac{1}{2}$. |
| " " | 12.00 " | " " |
| " 9th, | 5.30 A. M. | " " |
| " " | 9.00 " | Beef Tea, 4 ounces, by enema. |
| " " | 2.15 P. M. | Syr. Acacia, 2 drach., by mouth. |
| " " | 3.15 " | " " " |
| " " | 9.45 " | " " " |
| " " | 10.45 " | Beef Tea, 4 ounces, by enema. |
| " " | " " | Acet. Morph., gr. $\frac{1}{2}$, in Syr. Acacia. |
| " 10th, | 3.30 A. M. | Beef Tea, 3 ounces, by enema. |
| " " | 8.30 " | " " " |
| " " | 10.30 " | " 2 drach., by mouth. |
| " " | 11.30 " | " 3 " " |
| " " | 3.00 P. M. | " 1 ounce, " |
| " " | 8.30 " | " 1 " " |
| " 11th, | 9.00 A. M. | " 1 " " |
| " " | 10.00 " | " 1 " " |
| " " | 3.00 P. M. | " 1 " " |
| " " | 5.00 " | " 1 " " |
| " " | 7.00 " | " 1 " " |
| " 12th, | 9.00 A. M. | " 1 " and one egg. |
| " " | 1.00 P. M. | One egg. |
| " " | 6.00 " | Cream, half ounce. |

All of these prescriptions were administered in the presence of one of the surgeons in attendance; the time and quantities of the opiate being determined by the condition of the patient. Simple dressings were applied to the wound. Under this treatment, the progress of the case was perfectly satisfactory, and, at the date of the last prescription recorded, the patient was considered out of danger.

The first attempt to administer nourishment by the stomach, was made on the 8th, at 12.30 P. M., five days after the accident. One drachm of carefully prepared beef juice was then given. Some pain followed its exhibition. The same quantity was repeated two hours afterward, but with more pain. Another attempt was made in three hours more, but the pain was so severe we were obliged to desist, and to give a grain and a half Morphia in the course of the night. A little syrup of acacia was given the next afternoon. Some pain was complained of at first, but the succeeding portion gave less. On the 10th, the beef juice was again tried cautiously, and it was found the stomach would bear it. From this time, the diet was gradually improved, but solid food could not be borne for several weeks.

Seven weeks after the injury, I extracted the ball. It had lodged just under the integuments in the space between the angle of the ribs and spine on the left side, near the last dorsal vertebra. This wound remained open till the 15th May, 1855. There is now quite an enlargement of the wounded cartilage at its junction with the sternum and extending toward the left nipple. The patient has been a great sufferer, and has frequently been obliged to support himself by crutches in his walks. In January, 1856, when I last saw him, he was apparently in very good health and had almost regained his usual flesh. He tells me that after a full meal, he feels the stomach dragging upon the ribs, and is sure it is adherent to their inner surface.

This case affords an example of the great value of the *defensive* use of opium in lesions of the abdominal viscera, or wherever inflammation of the serous tissues is to be apprehended. Considering how illy prepared this patient's constitution was, to withstand so severe an injury, I think I am justified in believing that, without the prophylactic use of opium, gastritis and peritonitis must have supervened, and in all probability would have proved fatal. In similar cases, I think it will be found easier to anticipate these accidents by the free use of opium than to remedy them by the most judicious treatment after they shall have set in.

It is further to be observed in this case that, by the constant use of the Morphia, the patient never felt a sensation of hunger, and was thus enabled to bear the protracted abstinence required without any aggravation of the existing irritability of his system.—*Peninsular Journal of Medicine.*

